# Book Reviews

**Published:** 1911-08

**Authors:** 


### BOOK REVIEWS

THE PRINCIPLES AND PRACTICE OF MODERN OTOLOGY. By John F. Barnhill, M. D., Professor of Otology, Laryngology, and Rhinology, Indiana University School of Medicine; and Ernest De W. Wales, B. S., M. D., Clinical Professor of Otology, Laryngology and Rhinology, Indiana University School of Medicine. Second edition revised. Octavo of 598 pages, with 305 original illustrations, many in colors. Philadelphia and London: W. B. Saunders Company, 1911. Cloth, $5.50; Half Morocco, $7.00 net.

Among the changes in this edition are:—The examination of the function of the ear has been entirely rewritten; more extended statement regarding operative injury to the facial nerve; a description of the "Conservative" Radical Mastoid operation; part of the text relating to the symptoms, pathology, and treatment of Labyrinth Suppuration. The book is well written and well illustrated and deserves the favorable comments received in its first edition.

---

A TEXT-BOOK OF MEDICAL DIAGNOSIS. By James M. Anders, M. D., Professor of the Theory and Practice of Medicine and of Clinical Medicine, and L. Napoleon Boston, M. D., Adjunct Professor of Medicine, Medico-Chirurgical College, Philadelphia. Octavo of 1195, with 443 illustrations, 17 in colors. Philadelphia and London: W. B. Saunders Company, 1911. Cloth, $6.00 net; Half Morocco, $7.00 net.

In this large volume the writers have furnished the improved methods of recognizing diseased conditions in their varying phases. The information gained by following such works must enable the physician to treat much more successfully the patients under his care than could the doctor less familiar with the facts. Nearly every up-to-date method is carefully considered.

---

CARE OF THE BABY. A manual for mothers and nurses containing practical directions for management of infancy and childhood in health and disease. By J. P. Crozier Griffith, M. D., Clinical Prof., diseases of children in the Hospital of the University of Pennsylvania, 5th edition, thoroughly revised. P. W. B. Saunders & Co., Philadelphia.

The sustained demand for this work shows well its usefulness and value. It is a reliable guide for anxious mothers.

HIERONYMUS FRACASTOR'S SYPHILIS, FROM THE ORIGINAL LATIN. A Translation in prose of Fracastor's immortal poem. Printed on hand-made Imported paper; Library Binding. Crown Octavo. The Philmar Company, Medical Publishers, Fidelity Building, St. Louis, Mo., Price $2.00.

Few books in medicine are written in such a splendid style as is Frascastor's on Sythilis. The transtation has been well done.

---

PRACTICAL CYSTOSCOPY AND THE DIAGNOSIS OF SURGICAL DISEASES OF THE KIDNEYS AND URINARY BLADDER. By Paul M. Pilcher, M. D., Consulting Surgeon to the Eastern Long Island Hospital. Octavo of 398 pages, with 233 illustrations, 29 in colors. Philadelphia and London: W. B. Saunders Company, 1911. Cloth, $5.50 net.

The precision in diagnosis made possible by the appropriate use of the systoscate has revolutionized in the last 10 years, surgery of the genito-urinary surgery. This splendid book by Pilcher is the best and most extensive work yet published in English upon this subject. The entire field is thoroughly covered.

---

PELLAGRA. By Dr. A. Marie, Physician to the Asylums, Department of the Seine; Editor-in-Chief, Archives de Neurologie, and Director of the Laboratory of Pathological Psychology, Ecole dei Hautes Etudes, Paris. With Introductory Notes by Professor Lombroso. Authorized Translation from the French, by C. H. Lavinder, M. D., Passed Assistant U. S. Public Health and Marine Hospital Service, and J. W. Babcock, M. D., Physician and Superintendent State Hospital for the Insane, Columbia, S. C. With Additions, Ilustrations, Bibliography and Apendices. The State Company, Columbia, S. C.

The importance of this subject cannot be overestimated as it has become most prevalent in the South. Every physician should be conversant with the history and facts so well presented in this book.

---

WHAT TO EAT AND WHY. By G. Carroll Smith, M. D., of Boston, Mass. Octavo of 310 pages. Philadelphia and London. W. B. Saunders Company, 1911. Cloth, $2.50 net.

Smith has described clearly and briefly the fundamental elements of food and the principles underlying its use, the essential reasons why a change in diet is sometimes necessary and how such changes should be made.

---

STRUCTURE AND FUNCTIONS OF THE BODY A hand book of anatomy and physiology for nurses and others desiring a practical knowledge of the subject. By Annette Fiske, A. M., Graduate of the Waltham Training School for Nurses. 12 mo. of 221 pages, illustrated. Philadelphia and London. W. B. Saunders Company, 1911. Cloth, $1.25 net.

Although written especially for nurses, this book should prove of great value to the medical student and general practitioner.

---

PROGRESSIVE MEDICINE. Edited by Hobart A. Hare, and Leighton T. Applemann. Vol. II, June, 1911. Lea & Febiger, Philadelphia.

This excellent quarterly digest of advances, discoveries and improvements in the medical and surgical sciences is well up to its standard and reputation and as usual reflects in a condensed. readable form up-to-date work.

The present issue contains: Hernia, by W. B. Coley, M. D.; Surgery of the Abdomen exclusive of Hernia, by A. G. Gerster, M. D.; Gynecology, by J. G. Clark, M. D.; Diseases of the Blood, etc., by A. Stengel, M. D., and Ophthalmology, by E. Jackson, M. D.

### PPOMRT RELIEF IN SCIATIC PAIN.

In reporting his experience in the treatment of sciatica, Fred E. Daivs, M. D., writes as follows in Annas olf Gynecology: "I have been giving antikamnia and codeine tablets a thorough trial in the treatment of sciatica and I must say that my success has been phenomenal indeed. I have also induced two other physicians to give them a fair trial and their success equals or surpasses my own. I meet with many cases of sciatica and before adopting antikamnia and codeine tablets I used a great deal of opium and morphine to relieve the poin. Since then, I have not given either. One of my patients had been conned to bed for fithree weeks during her last attack of sciatica. I prescribed one antikamnia and codeine tablet every four hours and in forty-eight hours she was up and has not felt the pain since."

